# Electrodeposition of High-Surface-Area IrO_2_ Films on Ti Felt as an Efficient Catalyst for the Oxygen Evolution Reaction

**DOI:** 10.3389/fchem.2020.593272

**Published:** 2020-10-23

**Authors:** Yu Jin Park, Jooyoung Lee, Yoo Sei Park, Juchan Yang, Myeong Je Jang, Jaehoon Jeong, Seunghoe Choe, Jung Woo Lee, Jung-Dae Kwon, Sung Mook Choi

**Affiliations:** ^1^Surface Technology Division, Materials Center for Energy Department, Korea Institute of Materials Science, Changwon, South Korea; ^2^Department of Materials Science and Engineering, Pusan National University, Busan, South Korea

**Keywords:** wet etching, iridium oxide, oxygen evolution reaction, electrodeposition, surface area

## Abstract

Under acidic conditions, IrO_2_ exhibits high catalytic activity with respect to the oxygen evolution reaction (OER). However, the practical application of Ir-based catalysts is significantly limited owing to their high cost in addition to the scarcity of the metal. Therefore, it is necessary to improve the efficiency of the utilization of such catalysts. In this study, IrO_2_-coated Ti felt (IrO_2_/Ti) electrodes were prepared as high-efficiency catalysts for the OER under acidic conditions. By controlling the surface roughness of the Ti substrate via wet etching, the optimum Ti substrate surface area for application in the IrO_2_/Ti electrode was determined. Additionally, the IrO_2_ film that was electrodeposited on the 30 min etched Ti felt had a large surface area and a uniform morphology. Furthermore, there were no micro-cracks and the electrode obtained (IrO_2_/Ti-30) exhibited superior catalytic performance with respect to the OER, with a mass activity of 362.3 A ${\text{g}}_{\text{Ir}}^{-1}$ at a potential of 2.0 V (vs. RHE) despite the low Ir loading (0.2 mg cm^−2^). Therefore, this proposed strategy for the development of IrO_2_/Ti electrodes with substrate surface control *via* wet etching has potential for application in the improvement of the efficiency of catalyst utilization with respect to the OER.

## Introduction

Hydrogen, which is a pollution-free energy resource with the convenience of long-term storage in small and large quantities without significant loss, has a number of distinct advantages as an alternative to fossil fuels (Bensaid et al., 2012; Nam et al., 2019). As a promising hydrogen production strategy, water electrolysis has emerged as a sustainable and eco-friendly technology (Brillet et al., 2012; Park et al., 2019b). Despite these benefits, one key reason it has not been utilized in practical applications is the slow associated rate of the oxygen evolution reaction (OER) (Zhou et al., 2020). The OER involves four electron-proton coupled reactions, and requires the use of a relatively higher amount of energy (higher overpotential) compared to the hydrogen evolution reaction (HER), which is a typical two electron-transfer reaction (Suen et al., 2017; Jang et al., 2020). To overcome these limitations, studies have been conducted with the aim to develop electrocatalysts to ensure the efficiency of the OER (Choi et al., 2018). Ir or Ru based catalysts demonstrate excellent catalytic properties with respect to the OER; however, because they are noble metals, they tend to be more expensive and scarce (Guo et al., 2019; Park et al., 2020a). Conversely, non-noble-metal based OER electrocatalysts are less costly and more abundant. However, under acidic pH conditions they are susceptible to corrosion and often show poor catalytic activity (Jiang et al., 2019; Park et al., 2020b). Given that the economy of hydrogen production via electrolysis is primarily based on catalytic activity rather than on the cost of the catalyst employed (Kanan and Nocera, 2008; Dinca et al., 2010), noble-metal catalysts, such as Ir- or Ru-based catalysts, which are very rare, account for a high proportion of OER electrocatalysts (Smith et al., 2014). Thus, studies to investigate the efficient use of small amounts of these catalysts are necessary.

To prepare electrodes, the catalyst coating methods currently in use include powder coating (Su et al., 2011; Al-Shroofy et al., 2017), sputtering (Sapountzi et al., 2011; Xiaokai et al., 2019), and electrodeposition (Yagi et al., 2005; Lee et al., 2016). Powder coating can be applied to large area electrodes; however, this is associated with low conductivity, and causes significant catalyst loss. Further, the sputtering method is associated with high conductivity and offers the possibility of controlling the surface morphology of the electrode with great ease. Yet, its application results in poor uniformity when applied over a large surface area. This method can also result in the damage of the substrate owing to ion collision. In contrast, with the electrodeposition method, it is possible to coat a substrate with a uniform composition metal film to obtain a smooth surface (Maliar et al., 2019; Park et al., 2019a). Additionally, the electrodeposition method also enables low-cost deposition via direct growth, and it is associated with efficient catalyst utilization (Kim et al., 2004; Fouda-Onana et al., 2014). Therefore, this is used to prepare electrodes for electrochemical applications (Fan et al., 2018; Wang et al., 2018).

Substrate adhesion and surface area are important factors that influence the quality of the catalyst layer when electrodes are prepared using the electrodeposition (Chalker et al., 1991; Marzouk, 2003). Generally, the formation of the native oxide layer on the substrate owing to exposure to air increases contact resistance and decreases substrate adhesion, which can be enhanced by removing the native oxide layer (Bryce et al., 2010). To provide a corrosion-resistant protective film that inhibits substrate passivation and acts as a cost effective catalyst layer for the OER, Choe et al. (2018) proposed the use of electrodeposited IrO_2_. However, studies on substrate surfaces for the electrodeposited IrO_2_ catalyst are limited. Hence, further studies are required to ensure the efficient control of the substrate surface before the electrodeposition.

In this study, IrO_2_/Ti electrodes were prepared via the electrodeposition of an iridium catalyst layer on a strong Ti felt in an acidic condition. To control the Ti felt surface and establish the optimal surface area, wet etching was employed, and the performances of the prepared electrodes with respect to the OER in acidic condition were investigated.

## Materials and Methods

### Preparation of IrO_2_/Ti Electrodes Using Anodic Electrodeposition

To prepare the electrodeposition solution, 0.1 M iridium chloride (IrCl_4_·H_2_O) was dissolved in DI water and stirred for 30 min. Then, 40 mM oxalic acid [(COOH)_2_·2H_2_O] and 100 mM hydrogen peroxide (H_2_O_2_) were added, and the mixture was homogenized for 10 min. To adjust the pH to 10.5, 340 mM potassium carbonate (34.5% K_2_CO_3_) was added, followed by stirring for 3 days to ensure the stability of the prepared electrodeposition solution (Lee et al., 2015).

IrO_2_/Ti electrodes were then prepared *via* electrodeposition on a titanium mesh (Ti gauze 80 mesh, Alpha Aeaser), which served as the substrate. Prior to the electrodeposition, etching with 5 wt.% oxalic acid was performed to remove any oxide layer or impurities that could be present on the Ti felt. Then, to observe the changes on the surface of the IrO_2_/Ti electrode with respect to the etching time, etching was performed for 0, 10, 20, 30, and 40 min in the etchant at 95°C.

A three-electrode system was used for the anodic electrodeposition. The titanium mesh and the standard calomel electrode (SCE) were used as a counter and reference electrode, respectively, and IrO_2_ electrodeposition was performed using a potentiostat (VMP-3, Bio-Logic, France) *via* the application of a constant current density of 2.5 mA cm^−2^ to the Ti felt for 10 min in the IrO_2_ electrodeposition solution at 25°C. The reaction that took place in this anodic electrodeposition is as follows.

$$\begin{array}{l} {\left[\text{Ir}{\left(\text{COO}\right)}_{2}{\left(\text{OH}\right)}_{4}\right]}^{2-}\to \text{Ir}{\text{O}}_{2}+2\text{C}{\text{O}}_{2}+2{\text{H}}_{2}\text{O}+2{\text{e}}^{-} \end{array}$$

### Physicochemical Characterization

Field emission scanning electron microscopy (FE-SEM, JEOL, JSM-7001F) and optical microscopy (OM, MXG-2500REZ) were performed to observe the surface morphology of the prepared IrO_2_/Ti electrodes. X-ray diffractometry (XRD, D/MAX 2500, Rigaku) was performed to observe the crystallinity of the IrO_2_ layer. For the conditions for the XRD, a Cu target at 40 kV and 200 mA, with a 2θ angle in the range 30–80° and a scanning rate of 1° min^−1^, was used. Additionally, the crystal structure of the IrO_2_ film was analyzed *via* X-ray photoelectron spectroscopy (XPS, ECSA2000 VG Microtech) using an Al Kα (1486.6 eV) light source, and each peak was fitted with carbon (284.6 eV). Inductively coupled plasma-mass spectroscopy (ICP-MS, iCAP 6300 sermodero Ltd.) was employed to determine the loading mass of Ir.

### Electrochemical Characterization

For the electrochemical characterization of the IrO_2_/Ti electrode, linear sweep voltammetry (LSV) and stability tests were performed using a potentiostat (VMP-3, Bio-Logic, France). IrO_2_/Ti (1 cm x 1 cm), Ag/AgCl (saturated KCl), and Pt were used as the working, reference, and counter electrodes of the three-electrode system, respectively. A 0.1 M solution of HClO_4_ was used as the electrolyte, and the LSV was performed within a voltage range of 0.25–2.0 V (vs. RHE). Additionally, the stability tests were conducted via the application of a constant current of 10 mA cm^−2^ for ~800 min, and electrochemical impedance spectroscopy (EIS) was performed within a frequency range of 100–200 kHz at a potential of 1.34 V.

## Results and Discussion

**Figure 1:**
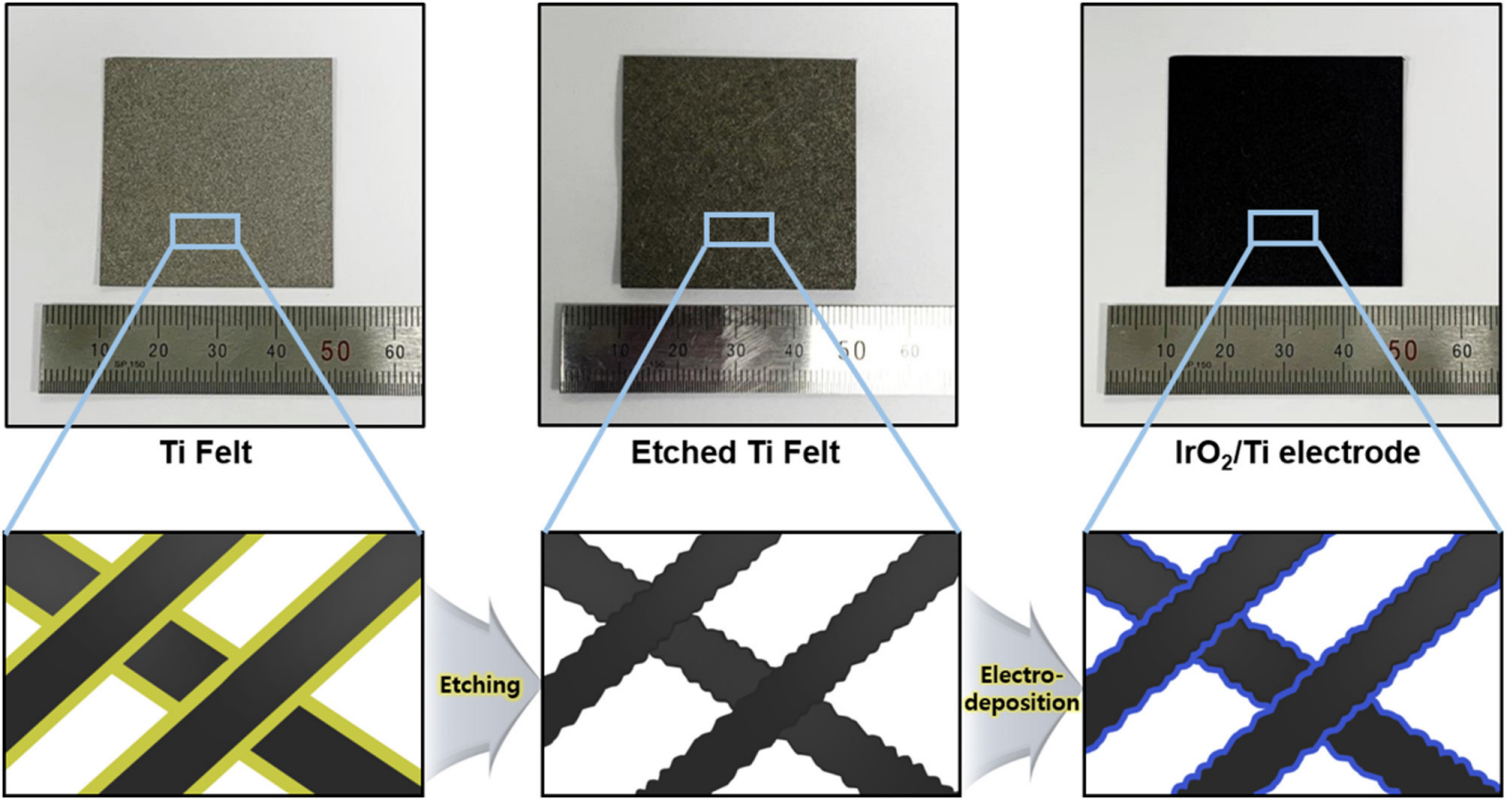
Schematic illustration of the IrO_2_/Ti electrode preparation process (Black, Ti wire; Yellow, native oxide layer; Blue, IrO_2_).

**Figure 2:**
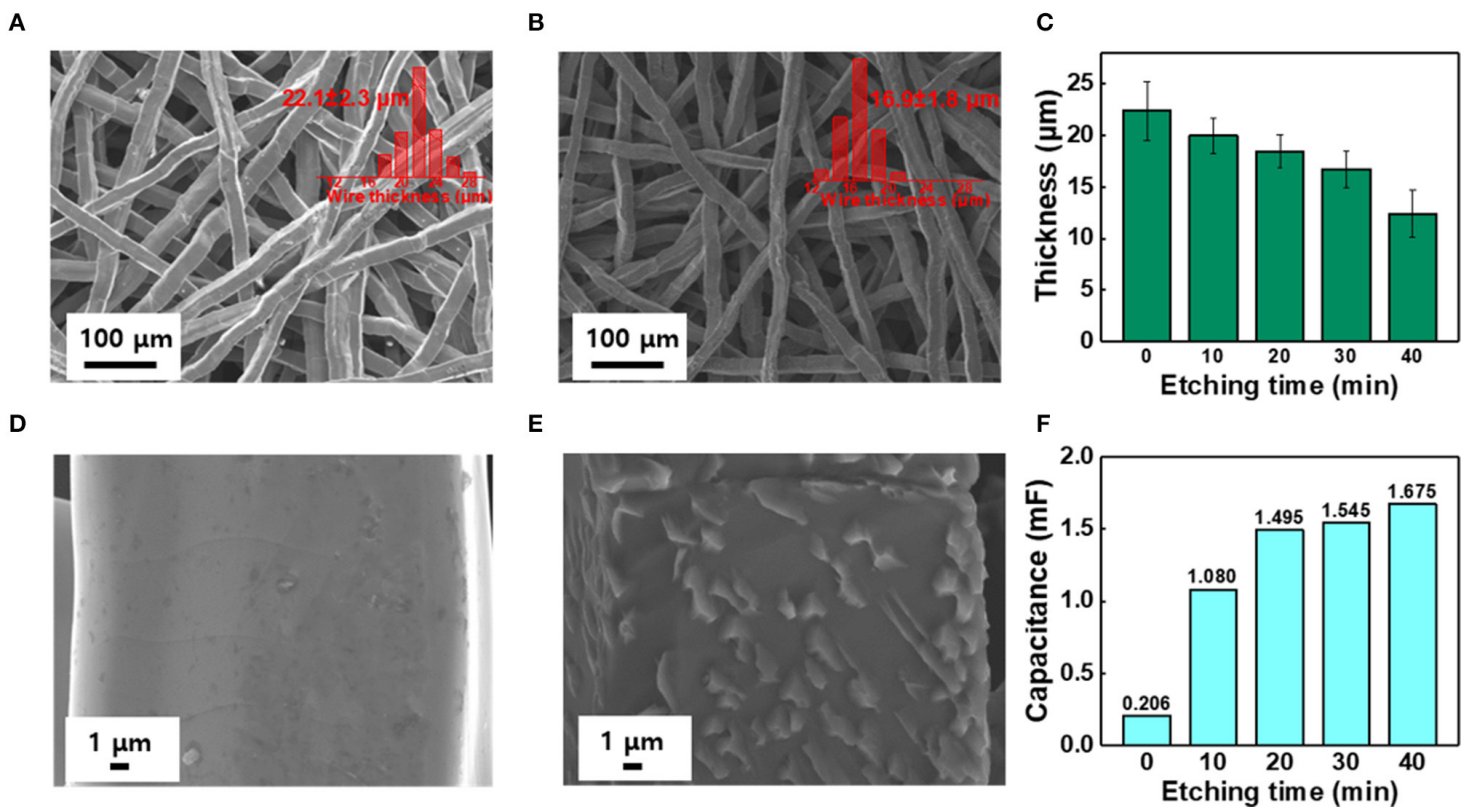
SEM image and wire thickness distribution (inset) of **(A,D)** Pristine Ti felt, and **(B,E)** The Ti felt etched for over 30 min. **(C)** Variation of Ti wire thickness with etching time. **(F)** Electrochemical double layer capacitance (C_dl_) analysis with respect to different etching times (0–40 min).

**Figure 3:**
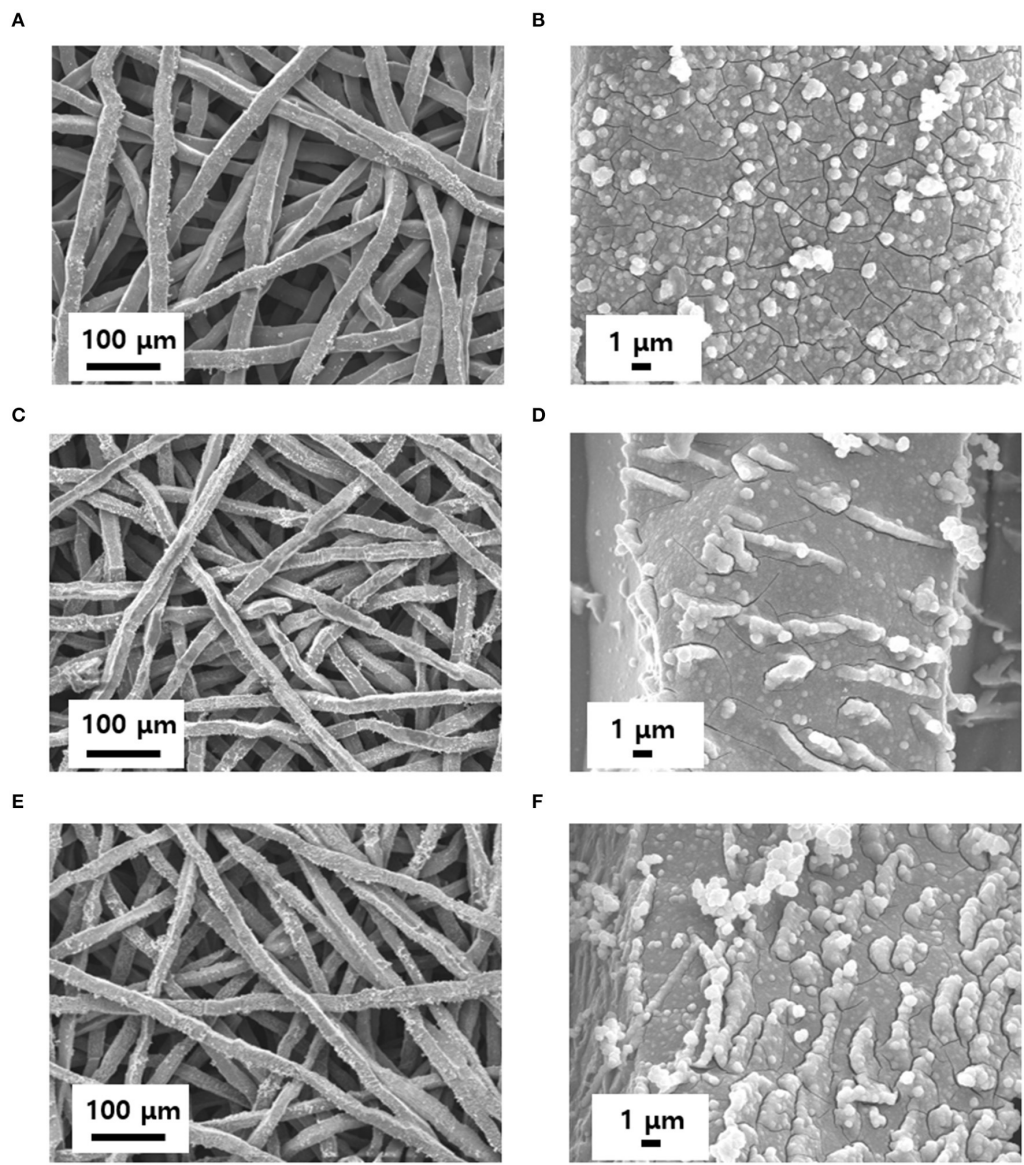
Evolution of IrO_2_/Ti morphology with time. The electrodes were prepared by controlling the etching time to: **(A,B)** 0, **(C,D)** 30, and **(E,F)** 40 min.

To remove the oxide layer on the surface of the Ti felt and control its roughness, the Ti felt substrate was etched in 5 wt.% oxalic acid, which served as the etchant. The etching time was varied at 0, 10, 20, 30, and 40 min, and the electrodes obtained were labeled IrO_2_/Ti- 0, 10, 20, 30, and 40, respectively. After etching, a layer of IrO_2_ was deposited on the Ti felt *via* the application of a current density of 2.5 mA cm^−2^ for 10 min at 25°C in an electrodeposition solution. Following the electrodeposition, the Ti felt was uniformly coated with the IrO_2_ film (Figure 1). The variation of the surface morphology of the Ti felt as a function of the etching time, which was observed using a field emission scanning electron microscope (FE-SEM), is shown in Figures 2A,B,D,E. The wire thickness of the Ti felt decreased from 22.1 ± 2.3 μm (etching time: 0 min) to 16.9 ± 1.8 μm (etching time: 30 min), and after etching for 40 min, it had decreased significantly (13.5 ± 1.7 μm), and became brittle (Figure 2C). Generally, the thickness of the native oxide layer of Ti is 3–7 nm (Supplementary Figure 1) (Wang et al., 2016). Given that the thickness of the Ti felt was reduced by more than ~2 μm as a result of etching, it was reasonable to consider that the native oxide layer had been completely removed. After etching for up to 20 min, the Ti felt displayed a smooth surface, which remained unchanged as the etching duration increased (Supplementary Figure 2). When the etching time increased above 30 min, the roughness of the Ti felt began to rise (Supplementary Figure 3). Figure 2F shows the electrochemical double-layer capacitance (C_dl_) value of the Ti felt with respect to the etching time. Generally, C_dl_ is proportional to the electrochemical surface area (ECSA), and is related to the roughness factors of the surface (Gira et al., 2016). As etching time increased, the C_dl_ value increased from 0.206 mF (0 min) to 1.675 mF (40 min). This indicates that there was an increase in the surface roughness in addition to the surface area of the Ti felt with the etching time (Supplementary Figure 4). The surface morphologies of the IrO_2_/Ti electrodes following electrodeposition are shown in Figure 3 and Supplementary Figure 5. The surface roughness of the Ti felt had an effect on the surface roughness of the electrodeposited IrO_2_ layer. The IrO_2_/Ti-0 electrode (Figures 3A,B) displayed cracks on the IrO_2_ layer that may be attributed to a high level of internal stress (Suvorov et al., 2018). Conversely, regarding the IrO_2_/Ti-30 electrode (Figures 3C,D), the coated IrO_2_ layer was flat and had minute visible cracks. This observation demonstrates the effect of chemical etching, and also indicates that an increase in etching time results in the removal of surface micro-cracks (Xiao et al., 2017). When the etching time was ≥40 min, the formation of IrO_2_ agglomerates around the surface roughness as a result of over-etching was observed (Figures 3E,F).

**Figure 4:**
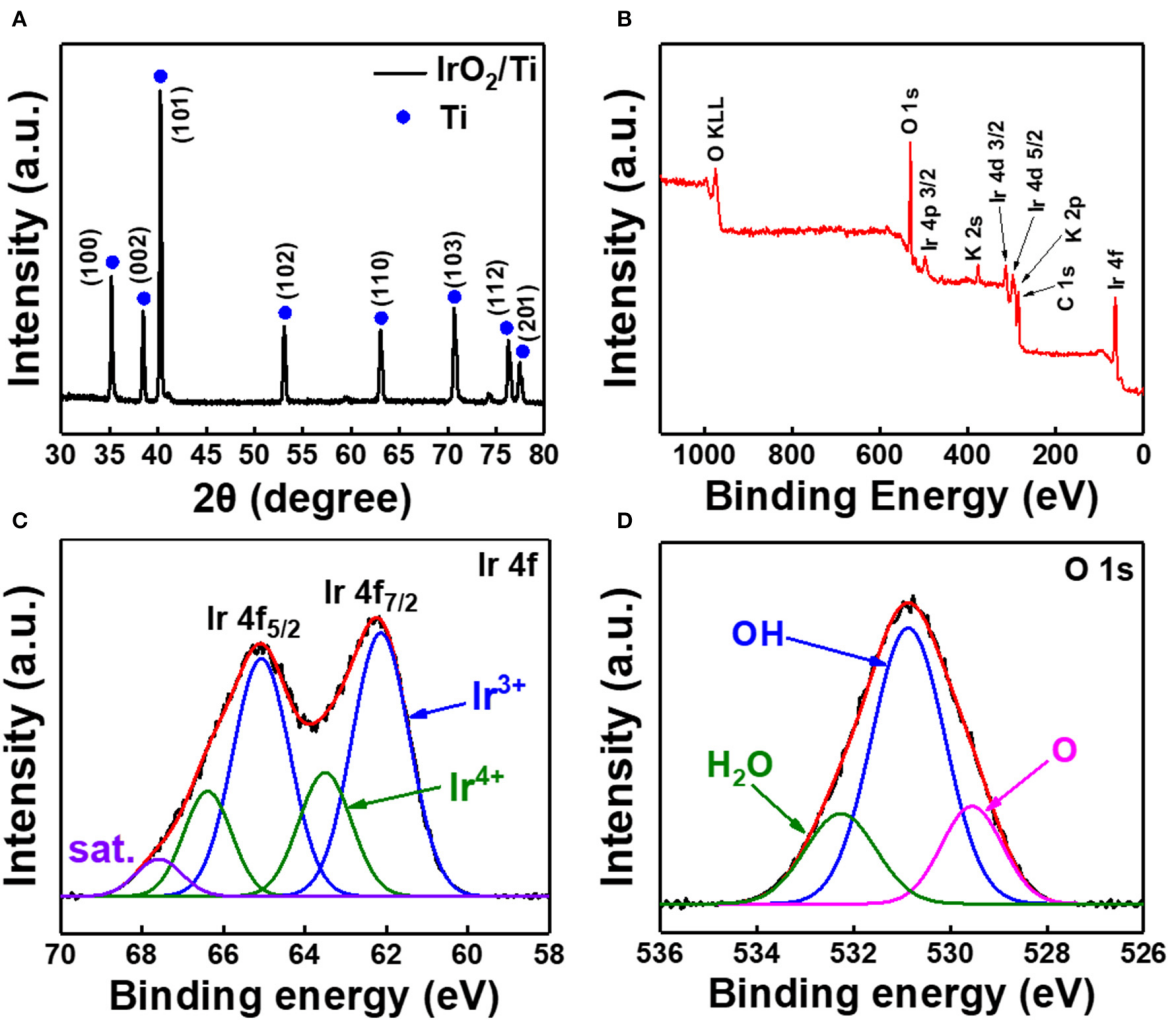
**(A)** X-ray diffraction pattern of IrO_2_/Ti-30. **(B)** Full profile XPS spectrum, **(C)** Ir 4f XPS spectrum, and **(D)** O 1s XPS spectrum of the IrO_2_/Ti-30 electrode.

The results of the analysis of the structure of the IrO_2_/Ti-30 electrode using XRD and X-ray photoelectron spectroscopy (XPS) are shown in Figure 4. Its XRD pattern showed no other peaks apart from those corresponding to Ti (Figure 4A). Figure 4B presented the full scan XPS spectra of IrO_2_/Ti-30 electrode. Additionally, its Ir 4f XPS spectra showed peaks at 65.1 eV (4f_5/2_) and 62.1 eV (4f_7/2_), which corresponded to the Ir^3+^ state, and others at 66.4 eV (4f_5/2_) and 63.5 eV (4f_7/2_), which corresponded to the unscreened component of the Ir^4+^ state (Figure 4C) (Choe et al., 2018). In the O 1s region of the high-resolution spectra of the IrO_2_/Ti-30 electrode, peaks corresponding to OH and O were observed at 530.9 and 529.6 eV, respectively (Figure 4D). Additionally, amorphous IrO_2_ demonstrated a higher hydroxide concentration than crystalline IrO_2_, indicating that the IrO_2_ layer on the Ti felt consisted of amorphous IrO_2_ (Jiang et al., 2019).

**Figure 5:**
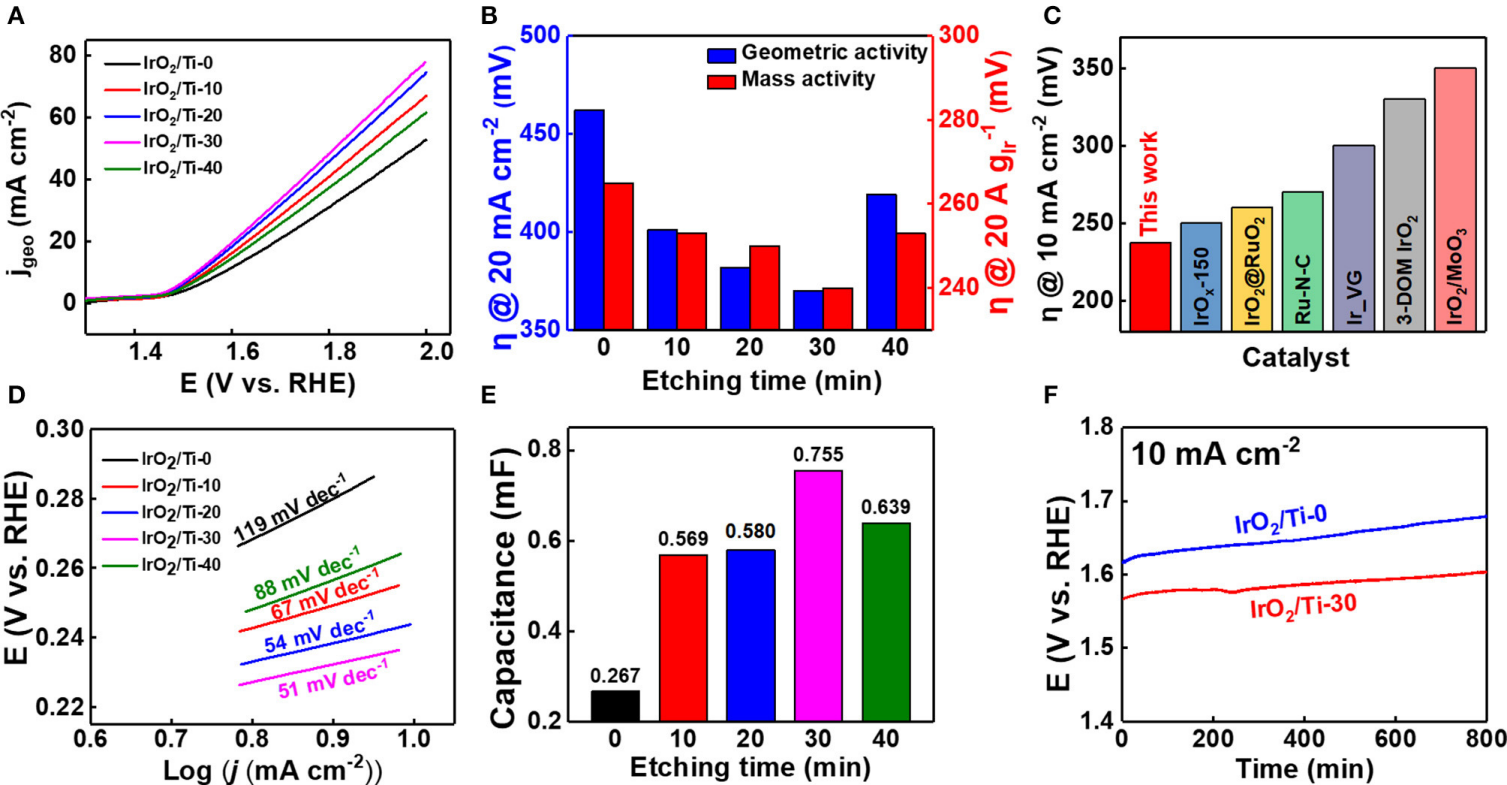
**(A)** Polarization curves of the IrO_2_/Ti-0, 10, 20, 30, 40 electrodes using 0.1 M HClO_4_ electrolyte without iR correction. **(B)** Bar graph showing the overpotential (η) of the geometric activity (20 mA cm^−2^) and mass activity (20 A ${\text{g}}_{\text{Ir}}^{-1}$). **(C)** Comparison of the OER overpotential (10 mA cm^−2^) obtained in this study with those of other noble metal electro catalyst in acidic condition with iR correction. **(D)** Tafel plots the electrodes with iR correction. **(E)** Variation of electrochemical double layer capacitance (C_dl_) with etching times (0–40 min). **(F)** Chronopotentiometry curves of the IrO_2_/Ti-30 (red) and IrO_2_/Ti-0 (blue) electrodes under a constant current density of 10 mA cm^−2^.

To evaluate the variation of the OER catalytic activity of the IrO_2_/Ti electrodes with etching time, electrochemical analyses were performed using 0.1 M HClO_4_ as the electrolyte, and the results obtained are illustrated in Figure 5. At 20 mA cm^−2^, the overpotential values corresponding to IrO_2_/Ti-0, IrO_2_/Ti-10, IrO_2_/Ti-20, IrO_2_/Ti-30, and IrO_2_/Ti-40, were 462, 401, 382, 370, and 419 mV, respectively (Figure 5A). Thus, the IrO_2_/Ti-30 electrode showed the highest OER activity. To determine the effect of etching time on the Ir loading mass, ICP-MS was performed. Observations revealed that as the etching time increased, the Ir loading mass increased from 0.183 mg cm^−2^ (IrO_2_/Ti-0) to 0.234 mg cm^−2^ (IrO_2_/Ti-20) (Supplementary Figure 6). After 30 min of etching, the loading mass began to decrease, dropping to 0.198 mg cm^−2^ (IrO_2_/Ti-40) at 40 min. Based on these Ir loading masses, the overpotential was recorded at 20 A ${\text{g}}_{\text{Ir}}^{-1}$ of mass activity (Figure 5B). The IrO_2_/Ti-30 electrode also showed the lowest overpotential value at 240 mV, and a comparison of the Ir mass activity at 2.0 V (vs. RHE), revealed the best OER performance (Supplementary Figure 7). When the IrO_2_ loading was excessive, some of the internal surfaces of the IrO_2_ layer could not be fully utilized (Zhang et al., 2020); thus, the IrO_2_/Ti-30 electrode showed superior catalytic performance relative to the IrO_2_/Ti-20 electrode, which had the largest IrO_2_ loading mass. The overpotentials shown in the iR-corrected LSV curve of the IrO_2_/Ti-30 electrode were compared with other noble metal electrocatalysts under acidic pH conditions (Supplementary Figure 8). The results obtained demonstrated that the IrO_2_/Ti-30 electrode had the lowest overpotential (Figure 5C and Supplementary Table 1). The Tafel slope derived from the iR-corrected LSV curve, which was calculated according to Equation (1), is shown in Figure 5D.

(1) $$\eta =A\;\times \;log\left(\frac{𝔦}{{𝔦}_{0}}\right)$$

where η, i, i_0_, and *A* represent the overpotential, current density, exchange current density, and the Tafel slope, respectively (Fang and Liu, 2014). The Tafel slope of the IrO_2_/Ti-30 electrode was 51 mV dec^−1^, which is lower than those of IrO_2_/Ti-0 (119 mV dec^−1^), IrO_2_/Ti-10 (67 mV dec^−1^), IrO_2_/Ti-20 (54 mV dec^−1^), and IrO_2_/Ti-40 (88 mV dec^−1^), indicating that the IrO_2_/Ti-30 electrode surface results in faster reaction rates in the OER. Hence, this demonstrates superior catalytic activity. ECSA enhancement implies an increase in the number of accessible Ir active sites (Zhang et al., 2020). Therefore, electrodes with large ECSA tend to exhibit superior catalytic activity. The capacitance value (C_dl_) of the IrO_2_/Ti-30 electrode, which is proportional to ECSA, was 0.755 mF. This C_dl_ value was higher than those of IrO_2_/Ti-0, IrO_2_/Ti-10, IrO_2_/Ti-20, and IrO_2_/Ti-40, which were 0.267, 0.569, 0.580, and 0.639 mF, respectively (Figure 5E and Supplementary Figure 9). Thus, it was reasonable to consider that the increase in ECSA could be attributed to the increase in surface roughness in the IrO_2_/Ti-30 electrode, leading to an enhancement of OER activity. When examining the IrO_2_/Ti-40 electrode, there was an increase in surface roughness, however, the formation of agglomerates on the surface, as shown in its SEM image, resulted in a decrease in specific surface area. This in turn resulted in a decrease in catalytic activity with respect to the OER (Hara et al., 2018).

The enhanced OER activity of the amorphous IrO_2_/Ti-30 electrode was attributed to its high surface area (Bernicke et al., 2015). The superior OER activity of the IrO_2_/Ti-30 electrode could also be attributed to the large amount of iridium hydroxide its surface. Under acidic conditions, the hydroxide of the amorphous IrO_2_ surface can react to form electrophilic O^I−^ species (IrO^x^O^II−^H ⇌ IrO^x^O^I−^ + H^+^+ e^−^). These species are susceptible to attack by water molecules or OH species; thus, the potential-determining step and rate-limiting step of the OER, resulting in the formation of O-O bonds, can be accelerated (Jiang et al., 2019). Therefore, the presence of hydroxide species on the surface of the IrO_2_ layer lower the adsorption energy required to attract H_2_O molecules. This leads to the accumulation of oxidation equivalents, which are associated with enhanced OER activity, at reaction sites (Bergmann et al., 2015). The study of the stability of the IrO_2_ catalyst layers revealed that the IrO_2_/Ti-30 electrode showed the highest OER activity and was evaluated using 0.1 M HClO_4_ as electrolyte at 25°C. The variation of its potential with time owing to the application of a constant current density of 10 mA cm^−2^ is shown in Figure 5F. After ~800 min, it was observed that its stability was higher than that of the IrO_2_/Ti-0 electrode. This superior stability could be attributed to the IrO_2_ catalyst layer, which serves as a passivation layer that protected the Ti felt from corrosion.

## Conclusion

In this study, an electrode for the OER was prepared via the electrodeposition of an amorphous IrO_2_-based catalyst on Ti felt. To increase the surface area of the Ti felt before the electrodeposition, its surface roughness was increased *via* wet etching. Thus, after etching for 30 min, there was an increase in its surface roughness, and the amorphous IrO_2_ layer was uniformly deposited. This resulted in superior OER catalytic activity in comparison with the deposition of the crystalline phase, owing to the presence of surface iridium hydroxide. Additionally, the IrO_2_/Ti-30 electrode showed superior OER catalytic activity and mass activity, which may be attributed to an increase in active sites owing to an increase in surface roughness. Stability tests that lasted ~800 min also confirmed its excellent stability in comparison with the IrO_2_/Ti-0 electrode. This electrode with increased surface roughness and excellent stability, prepared via electrodeposition and etching, demonstrated superior OER catalytic performance under acidic conditions. Therefore, the results of this study can serve as a promising path for the production of high-performance and low-cost OER electrodes for application in proton exchange membrane water electrolysis.

## Data Availability Statement

All datasets generated for this study are included in the article/Supplementary Material.

## Author Contributions

YJP and JL conceived the idea and designed the experiments. YJP, YSP, and SC synthesized the electrocatalysts and evaluated their electrochemical properties. JY and MJ performed the structure analysis. JL and JJ performed the physical characterizations. JWL, J-DK, and SMC coordinated and supervised the overall project. All authors reviewed the final manuscript.

## Conflict of Interest

The authors declare that the research was conducted in the absence of any commercial or financial relationships that could be construed as a potential conflict of interest.
