# Supplementary material for: Electrodeposition of High-Surface-Area IrO2 Films on Ti Felt as an Efficient Catalyst for the Oxygen Evolution Reaction
Source: Front Chem. 2020 Oct 23;8:593272. doi: 10.3389/fchem.2020.593272 (PMC7645052; doi:10.3389/fchem.2020.593272)
Supplement: Supplementary file 1 [file Data_Sheet_1.docx]

Supplementary Material

Electrodeposition of high-surface-area IrO_2_ films on Ti felt as efficient catalyst for the oxygen evolution reaction

Yu Jin Park^1,2†^, Jooyoung Lee^1†^, Yoo Sei Park^1,2^, Juchan Yang^1^, Myeong Je Jang^1^, Jaehoon Jeong^1^, Seunghoe Choe^1^, Jung Woo Lee^2*^_­­_, Jung-Dae Kwon^1*^, Sung Mook Choi^1*^

^1^ Materials Center for Energy Department, Surface Technology Division, Korea Institute of Materials Science, Changwon, 642831, Republic of Korea

^2^ Department of Materials Science and Engineering, Pusan National University, Busan 46241, Republic of Korea

*** Correspondence:**Jung Woo Lee
jungwoolee@pusan.ac.kr

Jung-Dae Kwon
jdkwon@kims.re.kr

Sung Mook Choi
akyzaky@kims.re.kr

# Supplementary Figures and Tables

## Supplementary Figures


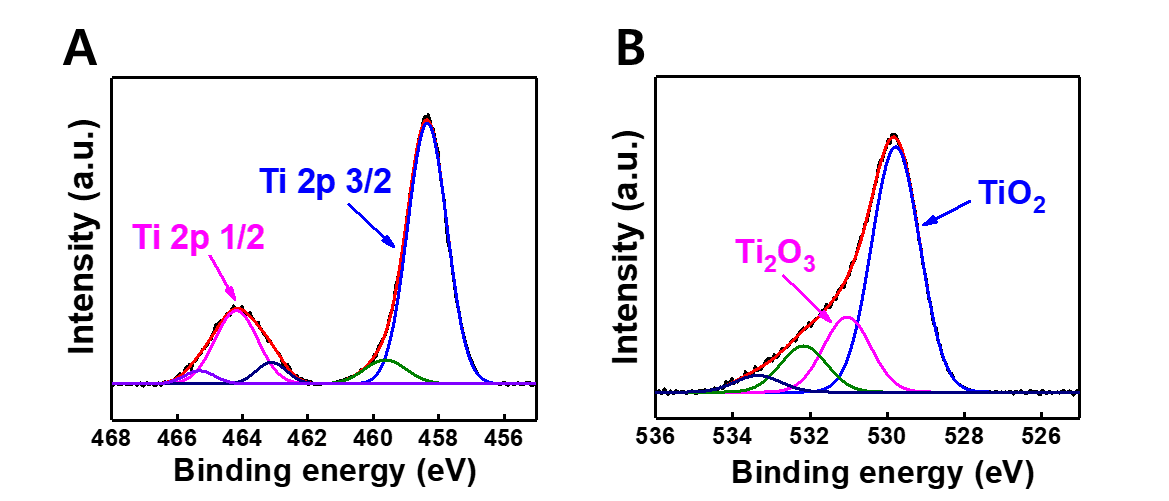


**Figure S1.** (**A**) Ti 2p XPS spectrum, and (**B**) O 1s XPS spectrum of the Ti felt.

**
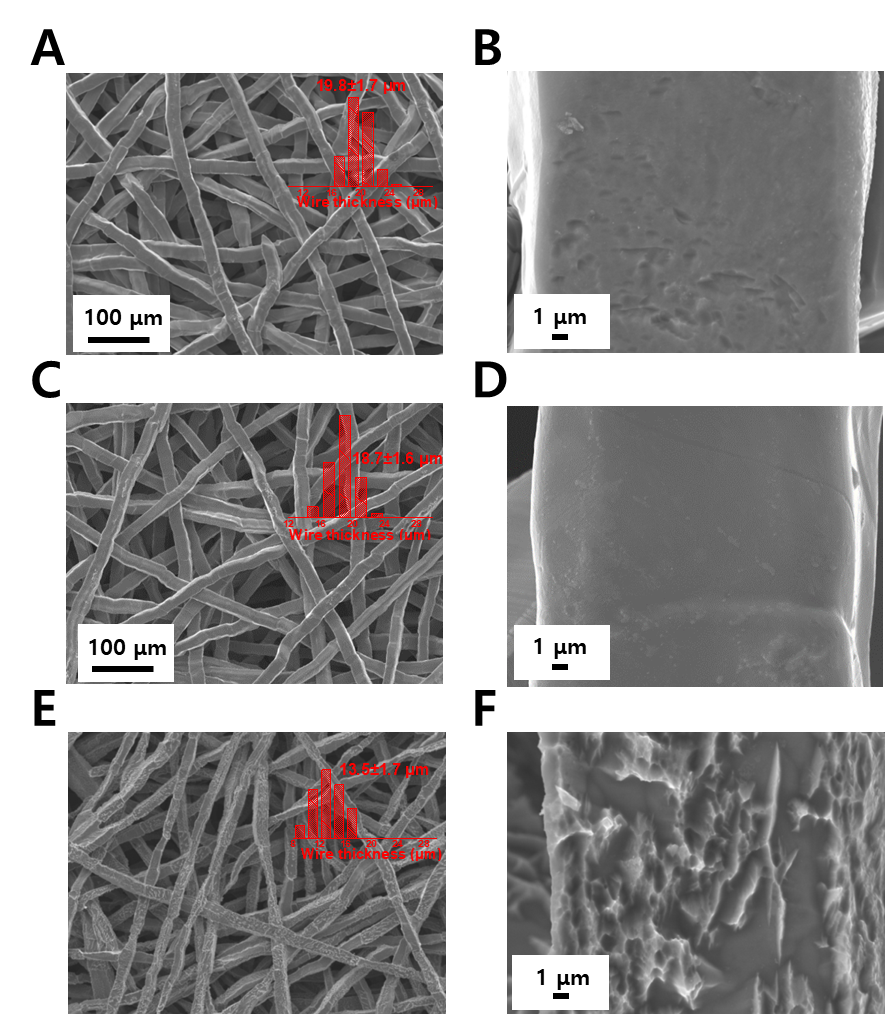
**

**Figure S2.** SEM image and wire thickness distribution (inset) of the etched Ti felt at: (**A**) and (**B**) 10, (**C**) and (**D**) 20, and (**E**) and (**F**) 40 min.


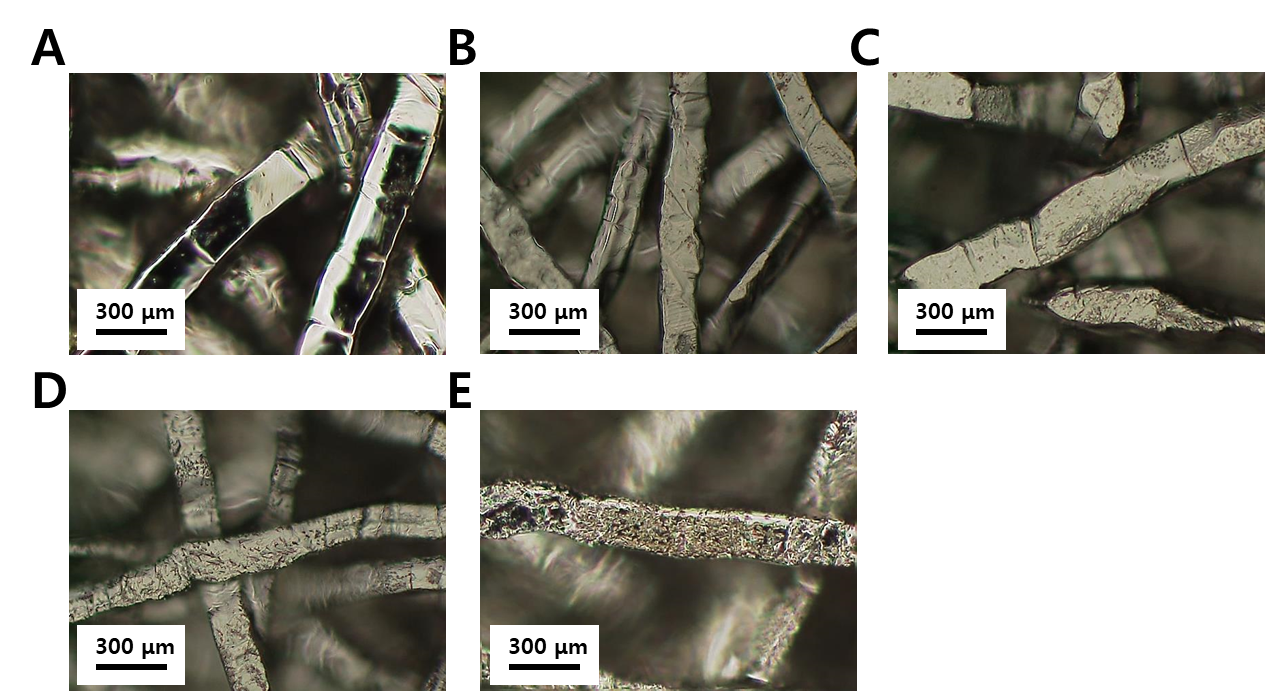


**Figure S3.** OM image of the etched Ti felt at: (**A**) 0, (**B**) 10, (**C**) 20, (**D**) 30, and (**E**) 40 min.


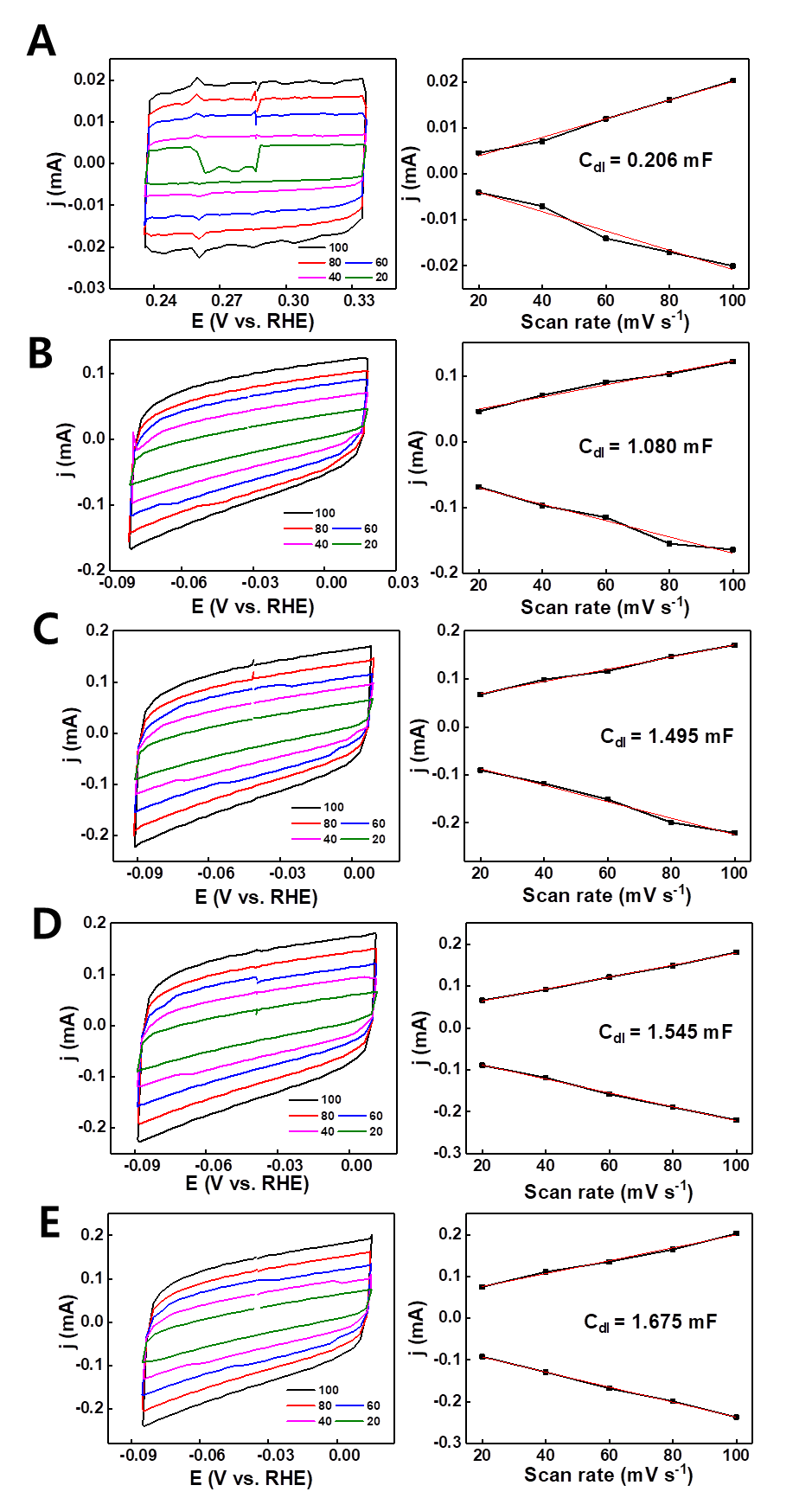


**Figure S4.** Cyclic voltammograms obtained at scan rates in the range 20–100 mV/s and the corresponding j, and scan rate plots for the Ti felt at etching times of: (**A**) 0, (**B**) 10, (**C**) 20, (**D**) 30, and (**E**) 40 min.


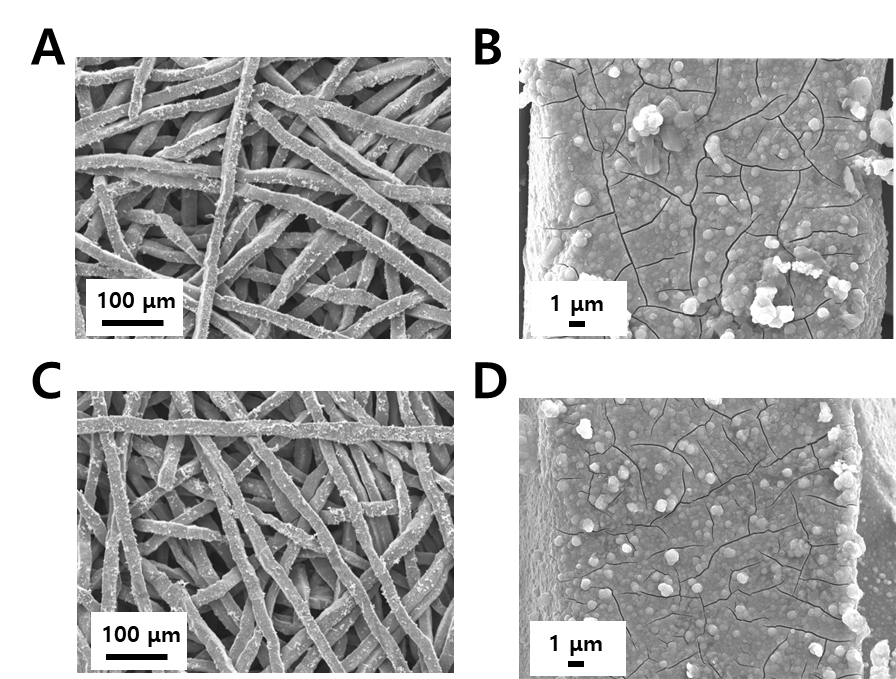


**Figure S5.** Time-evolution of the morphology of IrO_2_/Ti. The electrodes were prepared by controlling etching time to: (**A**), (**B**) 10, and (**C**), (**D**) 20 min.


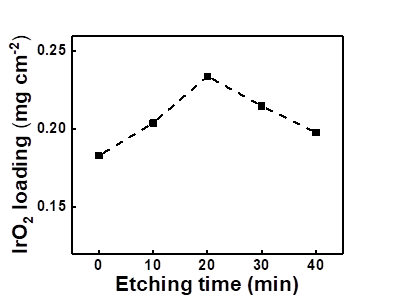


**Figure S6.** IrO_2_ loading as function of etching time.


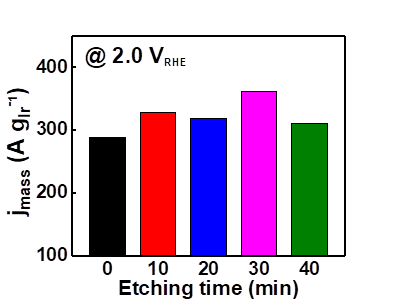


**Figure S7.** Iridium mass activity at 2.0 V (vs. RHE).


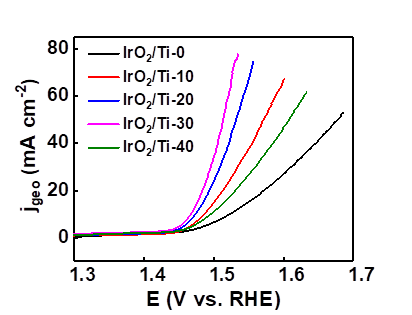


**Figure S8.** Polarization curves for the IrO_2_/Ti-0, 10, 20, 30, and 40 electrodes with iR correction using 0.1 M HClO_4_ as electrolyte.


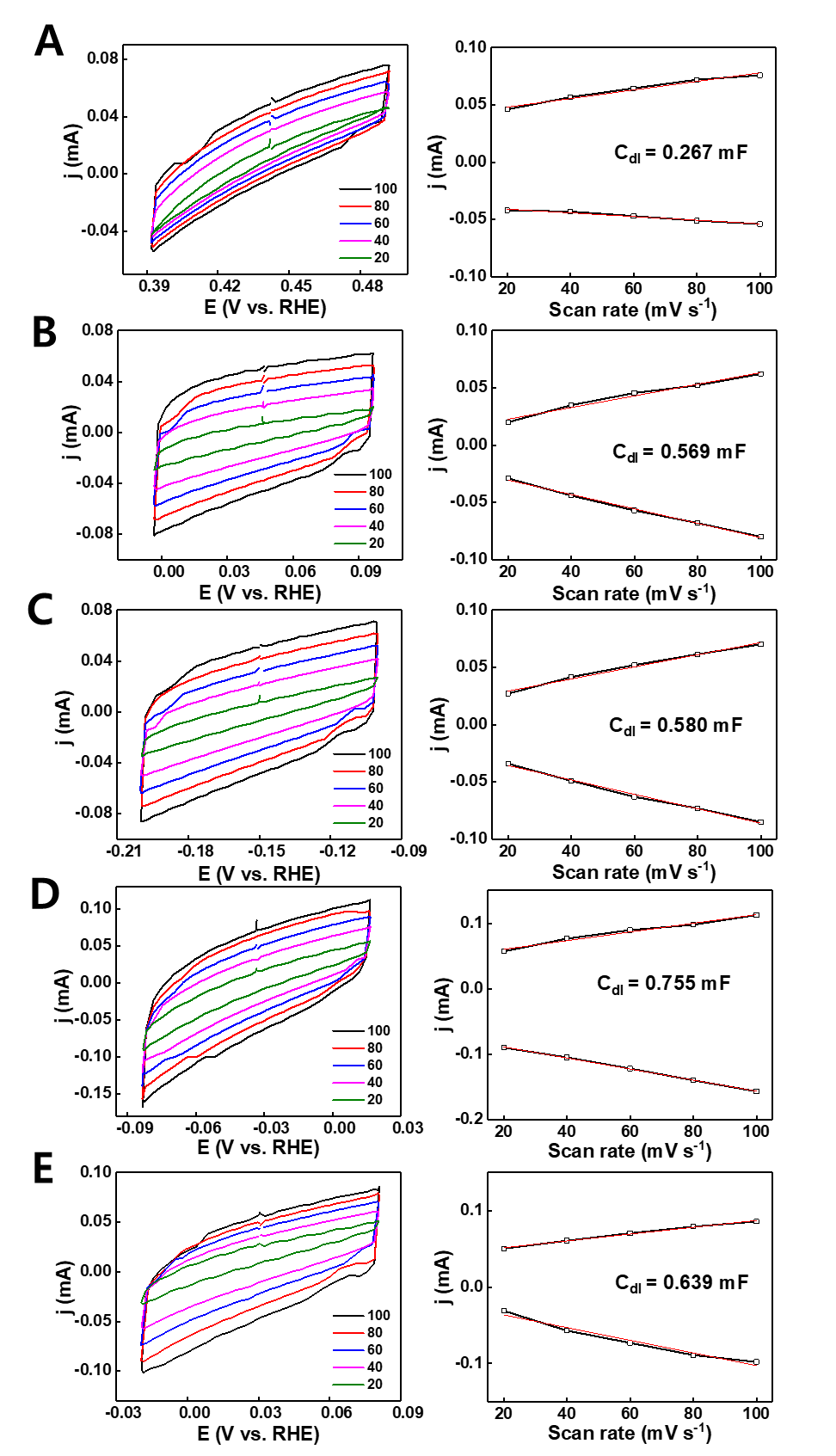


**Figure S9.** Cyclic voltammograms obtained at scan rates in the range 20–100 mV/s and the corresponding j, and the scan rates plots for: (**A**) IrO_2_/Ti-0, (**B**) IrO_2_/Ti-10, (**C**) IrO_2_/Ti-20, (**D**) IrO_2_/Ti-30, and (**E**) IrO_2_/Ti-40.

## Supplementary Tables

**Table S1.** Comparison of the OER activity of the IrO_2_/Ti-30 electrode with those of other noble metal electrocatalysts reported in literature, showing the overpotential at 10 mA cm^-2^ and the Tafel slope.

| Catalysts | η at 10 mA cm^-2^  (mV) | | Tafel slope  (mV dec^-1^) | Loading mass | Electrolyte | Ref. |
| --- | --- | --- | --- | --- | --- | --- |
| Electrodeposited amorphous IrO_2_/Ti-30 | | 237 | 51 | 0.2㎎ | 0.1 M HClO_4_ | This work |
| IrO_x_-150 | | 250 | 47 | 7.8 $㎍$ | 0.5 M H_2_SO_4_ | (Jiang et al. 2019) |
| IrO_2_@RuO_2_ | | 260 | 60 | - | 0.5 M H_2_SO_4_ | (Audichon et al. 2016) |
| Ru-N-C | | 270 | 52.6 | 1.0 wt.% | 0.5 M H_2_SO_4_ | (Cao et al. 2019) |
| Ir_VG | | 300 | 59 | - | 0.5 M H_2_SO_4_ | (Roy et al. 2019) |
| 3-DOM IrO_2_ | | 330 | ­- | 127$㎍$ | 0.5 M H_2_SO_4_ | (Hu et al. 2012) |
| IrO_2_/MoO_3_ | | 350 | 57 | 0.2㎎ | 0.1 M HClO_4_ | (Tariq et al. 2018) |

**References**

Audichon, T., Napporn, T. W., Canaff, C., Morais, C., Comminges, C., Kokoh, K. B. (2016). 'IrO2 Coated on RuO_2_ as Efficient and Stable Electroactive Nanocatalysts for Electrochemical Water Splitting'. *J. Phys. Chem. C* 120(5), 2562–2573. doi:[10.1021/acs.jpcc.5b11868](https://sci-hub.tw/10.1021/acs.jpcc.5b11868).

Cao, L., Luo, Q., Chen, J., Wang, L., Lin, Y., Wang, H. et al. (2019). 'Dynamic oxygen adsorption on single-atomic Ruthenium catalyst with high performance for acidic oxygen evolution reaction'. *Nat. Commun.* 10(1), 4849. doi:[10.1038/s41467-019-12886-z](https://sci-hub.tw/10.1038/s41467-019-12886-z).

Hu, W., Wang, Y., Hu, X., Zhou, Y., Chen, S. (2012). 'Three-dimensional ordered macroporous IrO_2_ as electrocatalyst for oxygen evolution reaction in acidic medium'. *J. Mater. Chem.* 22(13), 6010. doi:[10.1039/C2JM16506F](https://sci-hub.tw/10.1039/C2JM16506F).

Jiang, B., Kim, J., Guo, Y., Wu, K. C. W., Alshehri, S. M., Ahamad, T. et al. (2019). ‘Efficient oxygen evolution on mesoporous IrO_x_ nanosheets’. *Catal. Sci. Technol.* 9, 3697–3702. doi:[10.1039/C9CY00302A](https://doi.org/10.1039/c9cy00302a).

Roy, S. B., Akbar, K., Jeon, J. H., Jerng, S.-K., Truong, L., Kim, K. et al. (2019). 'Iridium on vertical graphene as an all-round catalyst for robust water splitting reactions'. *J. Mater. Chem. A 7, 20590*–*20596.* doi:[10.1039/c9ta07388d](https://sci-hub.tw/10.1039/c9ta07388d).

Tariq, M., Zaman, W. Q., Sun, W., Zhou, Z., Wu, Y., Cao, L. et al. (2018). 'Unraveling the Beneficial Electrochemistry of IrO_2_/MoO_3_ Hybrid as a Highly Stable and Efficient Oxygen Evolution Reaction Catalyst'. *ACS Sus. Chem. Eng.* 6(4), 4854–4862. doi:[10.1021/acssuschemeng.7b04266](https://sci-hub.tw/10.1021/acssuschemeng.7b04266).
